# Exercise‐induced improvement of glycemic fluctuation and its relationship with fat and muscle distribution in type 2 diabetes

**DOI:** 10.1111/1753-0407.13549

**Published:** 2024-04-07

**Authors:** Dan Liu, Ying Zhang, Qian Wu, Rui Han, Di Cheng, Liang Wu, Jingyi Guo, Xiangtian Yu, Wenli Ge, Jiacheng Ni, Yaohui Li, Tianshu Ma, Qichen Fang, Yufei Wang, Yan Zhao, Yanan Zhao, Biao Sun, Huating Li, Weiping Jia

**Affiliations:** ^1^ Department of Endocrinology and Metabolism Shanghai Sixth People's Hospital Affiliated to Shanghai Jiao Tong University School of Medicine, Shanghai Diabetes Institute, Shanghai Clinical Center for Diabetes, Shanghai Key Laboratory of Diabetes Mellitus Shanghai China; ^2^ Clinical Research Center Shanghai Sixth People's Hospital Affiliated to Shanghai Jiao Tong University School of Medicine Shanghai China; ^3^ School of Sports Science and Physical Education Nanjing Normal University Nanjing China; ^4^ Department of Kinesiology Nanjing Sport Institute Nanjing China; ^5^ Department of Sports and Health Science Nanjing Sport Institute Nanjing China

**Keywords:** body composition, continuous glucose monitoring, diabetes, exercise, glycemic variability, magnetic resonance imaging

## Abstract

**Aims:**

Management of blood glucose fluctuation is essential for diabetes. Exercise is a key therapeutic strategy for diabetes patients, although little is known about determinants of glycemic response to exercise training. We aimed to investigate the effect of combined aerobic and resistance exercise training on blood glucose fluctuation in type 2 diabetes patients and explore the predictors of exercise‐induced glycemic response.

**Materials and Methods:**

Fifty sedentary diabetes patients were randomly assigned to control or exercise group. Participants in the control group maintained sedentary lifestyle for 2 weeks, and those in the exercise group specifically performed combined exercise training for 1 week. All participants received dietary guidance based on a recommended diet chart. Glycemic fluctuation was measured by flash continuous glucose monitoring. Baseline fat and muscle distribution were accurately quantified through magnetic resonance imaging (MRI).

**Results:**

Combined exercise training decreased SD of sensor glucose (SDSG, exercise‐pre vs exercise‐post, mean 1.35 vs 1.10 mmol/L, *p* = .006) and coefficient of variation (CV, mean 20.25 vs 17.20%, *p* = .027). No significant change was observed in the control group. Stepwise multiple linear regression showed that baseline MRI‐quantified fat and muscle distribution, including visceral fat area (β = −0.761, *p* = .001) and mid‐thigh muscle area (β = 0.450, *p* = .027), were significantly independent predictors of SDSG change in the exercise group, as well as CV change.

**Conclusions:**

Combined exercise training improved blood glucose fluctuation in diabetes patients. Baseline fat and muscle distribution were significant factors that influence glycemic response to exercise, providing new insights into personalized exercise intervention for diabetes.

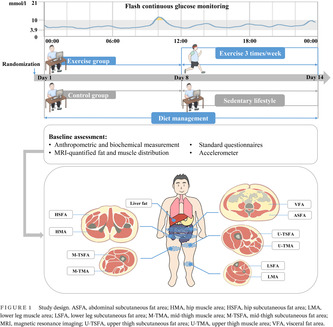

## Graphical abstract

Highlights

## 1 INTRODUCTION

The increasing prevalence of diabetes has become an emerging health challenge worldwide. According to the International Diabetes Federation, it was estimated to affect 537 million people in 2021, rising to 783 million by 2045.^1^ Diabetes is a chronic metabolic disease characterized by a series of complications, leading to increased disability and mortality,^2^ and greater global health expenditures.^1^ Glycemic control is essential for the prevention and management of diabetes and its complications.

Glycated hemoglobin (HbA1c) is the classic metric for evaluating overall glycemic control in clinical practice, but it does not provide information on the risk of hypoglycemia or glycemic variability.^3^, ^4^ Continuous glucose monitoring (CGM) systems, including real‐time CGM and flash CGM (FGM), provide a series of useful metrics for glycemic control, such as time in range (TIR), mean sensor glucose (MSG), SD of sensor glucose (SDSG), coefficient of variation (CV), and mean amplitude of glucose excursions.^5^ It is noteworthy that high glycemic variability has been shown to increase the risk of diabetic complications, even more than persistent hyperglycemia.^6^, ^7^, ^8^, ^9^

Exercise as an important therapeutic strategy for diabetes and its complications is generally accepted and recommended worldwide.^10^, ^11^, ^12^ Clinical studies have demonstrated that combined aerobic and resistance exercise training is more conducive to improve blood glucose control, lipid metabolism, and cardiopulmonary fitness.^13^, ^14^ Previous studies commonly focused on the effect of exercise on overall glycemic control, focusing on HbA1c and fasting plasma glucose (FPG). With the application of CGMs, increasing attention has been paid by researchers and clinicians to the effect of exercise on blood glucose fluctuation in diabetes patients.^15^, ^16^ Nevertheless, there are individual differences in response to regular exercise. Not all individuals benefit from exercise training: some display exercise resistance.^17^ So far, the underlying mechanisms, determinants, or predictors of exercise‐induced metabolic benefits remain unclear. Understanding why this metabolic heterogeneity exists is critical for further advances in personalized exercise prescription.

Type 2 diabetes patients have a body shape of abdominal obesity and small thigh circumference,^18^, ^19^ which becomes more obvious with age.^20^ Visceral fat participates in the pathophysiological mechanism of diabetes and its complications,^21^ and skeletal muscle is involved in the uptake of glucose and essential for regulating systemic metabolism.^22^ Exercise can reduce visceral fat and induce the adaptive response of skeletal muscle in patients with type 2 diabetes.^23^, ^24^ Nonetheless, whether the heterogeneity of body composition among diabetes patients partly determines the impact of exercise on blood glucose fluctuation is still uncertain.

In this study, we aimed to investigate the effect of combined exercise training on blood glucose fluctuation in patients with type 2 diabetes and explore the possible determinants of exercise response. FGM was applied to continuously monitor glucose concentrations. Moreover, the body compositions of participants were accurately quantified through magnetic resonance imaging (MRI), MRI‐proton density fat fraction (MRI‐PDFF), and bioelectrical impedance analysis (BIA). We hypothesized that exercise would improve glycemic variability and that baseline fat and muscle distribution would have a considerable impact on the glycemic response to exercise.

## 2 MATERIALS AND METHODS

### 2.1 Study design and participants

This study was a randomized controlled clinical trial, registered at Chinese Clinical Trial Registry (ChiCTR2100046148). The study was approved by the Ethics Committee of the Shanghai Sixth People's Hospital (2019–099). Written informed consents were obtained from all participants. The rationale, design, and methods of this study have been previously reported.^25^ Briefly, potential participants were recruited at the Shanghai Sixth People's Hospital Affiliated to Shanghai Jiao Tong University School of Medicine. Adults who were aged 35–65 years of any sex, sedentary, diagnosed with type 2 diabetes mellitus, abdominal obesity (waist circumference ≥90 cm for males and ≥80 cm for females), and not insulin dependent were included for eligibility assessment. Patients who met all the inclusion and exclusion criteria and completed the run‐in period were invited to this study, and then they were randomly allocated to either an exercise or a control group in a 1:1 ratio stratified by sex and age.

**FIGURE 1:**
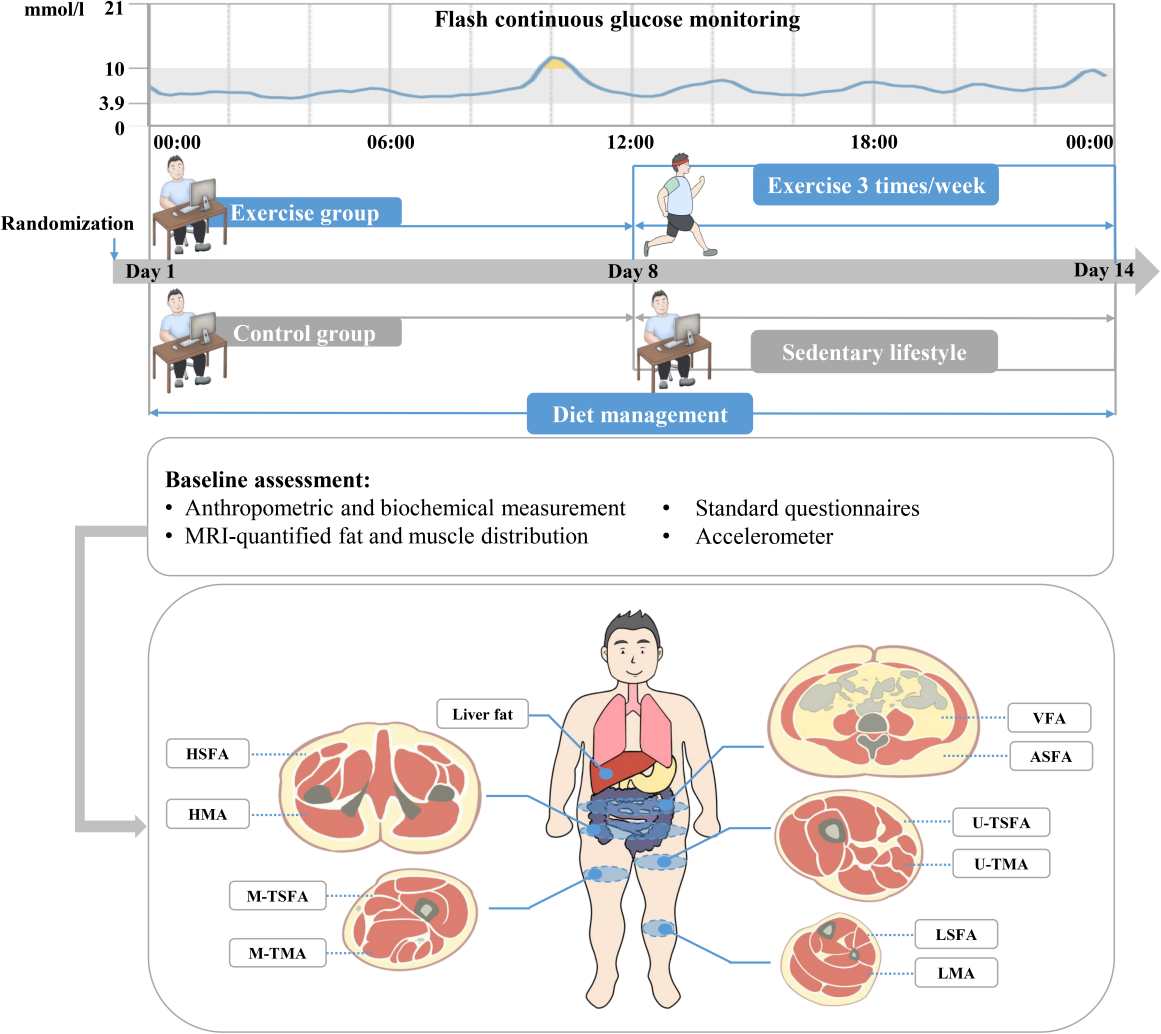
Study design. ASFA, abdominal subcutaneous fat area; HMA, hip muscle area; HSFA, hip subcutaneous fat area; LMA, lower leg muscle area; LSFA, lower leg subcutaneous fat area; M‐TMA, mid‐thigh muscle area; M‐TSFA, mid‐thigh subcutaneous fat area; MRI, magnetic resonance imaging; U‐TSFA, upper thigh subcutaneous fat area; U‐TMA, upper thigh muscle area; VFA, visceral fat area.

Figure 1 shows the study design. Participants in the control group maintained sedentary lifestyle for 2 weeks, while those in the exercise group maintained sedentary lifestyle for 1 week, and then received combined aerobic and resistance training three times a week. Additionally, all participants received dietary management according to the Dietary Guidelines for Chinese Residents^26^ and Dietary Guidelines for Type 2 diabetes in China^27^ (as detailed subsequently). Participants were asked to record the daily food intakes on the Boohee Nutrition Mini Program (Shanghai Mint Health Technology Co., Ltd, Shanghai, China) during the study. Their adherence to dietary regimens was assessed weekly by a nutritionist. The eligible diabetes patients participated in baseline assessment at the Department of Endocrinology and Metabolism, Shanghai Sixth People's Hospital Affiliated to Shanghai Jiao Tong University School of Medicine. Apart from the anthropometry and hematology examination, a series of clinical assessments were performed. Baseline fat and muscle distribution were accurately quantified using MRI and MRI‐PDFF, including liver fat, visceral fat area (VFA), subcutaneous fat area of abdomen (ASFA), hip (HSFA), upper thigh (U‐TSFA), mid‐thigh (M‐TSFA), and lower leg (LSFA), and muscle area of hip (HMA), upper thigh (U‐TMA), mid‐thigh (M‐TMA), and lower leg (LMA) (shown in Figure 1). Three‐dimensional accelerometer tester (wGT3x‐BT, Manufacturing Technology Inc, MTI, Florida, USA) was used to evaluate the amount of physical activity. FGM was used to evaluate the blood glucose fluctuation of participants for 14 consecutive days.

### 2.2 Study intervention

#### 2.2.1 Exercise intervention

Previous studies suggested that acute or short‐term exercise training could improve glucose control.^15^, ^16^, ^28^ Here, we designed the trial of combined exercise training, studied its effect on blood glucose levels recorded by FGM in patients with type 2 diabetes and further explored factors that influence glycemic response to exercise. Considering the heterogeneity in physical fitness levels among individuals, an individualized exercise training regimen was developed for each participant based on their cardiopulmonary fitness and one repetition maximum. Patients participated in combined exercise training sessions three times per week under the guidance of professional coaches at the Shanghai Sixth People's Hospital Affiliated to Shanghai Jiao Tong University School of Medicine. The training sessions were approximately 80 min, including warm‐up (10 min), aerobic exercise (30 min, 40% of maximum oxygen uptake), rest (10 min), resistance training (20 min, eight repetitions in two groups, 50% of one repetition maximum), and relaxation (10 min). Participants performed aerobic exercise on magnetic cycle ergometer and resistance exercise on the training machine for the muscle group in trunk and upper and lower limbs (Nanjing Kuanyue Health Technology Co., Ltd, Nanjing, China).^25^ The duration and intensity of training session were recorded. Meanwhile, the finger‐stick blood glucose, blood pressure, and heart rate of participants were recorded before and after the training sessions.

#### 2.2.2 Diet management

Diet management was conducted during the study intervention period to ensure similar dietary intake among participants. All participants were required to prepare and consume food based on a recommended dietary regimen, which was designed according to the Dietary Guidelines for Chinese Residents^26^ and Dietary Guidelines for Type 2 diabetes in China.^27^ The recommended daily energy intake was equal to 25 (kcal/kg) multiplied by ideal body weight (kg), which was calculated as the participant's height (cm) minus 105 (cm). The diets contained approximately 50% carbohydrate, 30% fat, and 20% protein. To increase the dietary compliance, participants were asked to record the type and volume of food that they consumed and were encouraged to take and upload photos of each meal on the Boohee Nutrition Mini Program (Shanghai Mint Health Technology Co., Ltd, Shanghai, China). A trained dietician assessed the food record every week to evaluate participants' adherence to the dietary regimens. Based on these records, the Boohee Nutrition Mini Program automatically calculated the intake of total energy, macronutrients, and other nutrients.

### 2.3 Clinical assessment

After overnight fasting for at least 10 h, participants received general information collection, anthropometric and biochemical measurements, and standard questionnaires at the Department of Endocrinology and Metabolism, Shanghai Sixth People's Hospital Affiliated to Shanghai Jiao Tong University School of Medicine. Blood pressure, height, weight, and waist, hip, and thigh circumference were measured. Body weight and height were measured without shoes and with light clothing. Blood pressure was measured twice after at least 5 min of sitting using a sphygmomanometer at 2‐ min intervals, and the average value was calculated. Waist circumference was measured at the midpoint between the inferior costal margin and the superior border of the iliac crest on the midaxillary line. Hip circumference was measured at the widest part over the greater trochanters. Thigh circumference was measured at the midpoint of the line between the anterior superior iliac spine and the upper edge of the patella. Body fat mass and percentage were measured using BIA (DBA‐210, software version 3.5, Donghuayuan Medical, Province Jilin, China).

Blood samples were collected in fasting state and then tested to measure HbA1c, FPG, fasting insulin (FINS), serum lipids, and liver and renal function. HbA1c was measured using high‐performance liquid chromatography (VARIANT II, Bio‐Rad Laboratories, Inc., Hercules, CA, USA). Plasma glucose concentrations were measured via glucose oxidase method. Insulin resistance and islet β cell function were evaluated by the homeostasis model assessment (HOMA): HOMA‐IR = FINS (mU/L) × FPG (mmol/L)/22.5, and HOMA‐β = [FINS (mU/L) × 6–3.33]/[FPG (mmol/L) −3.5].^29^

### 2.4 MRI‐quantified fat and muscle distribution

The 3.0 T MRI (Ingenia, Philips Medical System, the Netherlands) was used to scan the adipose tissue area of abdomen, hip, thigh, and lower leg, and the muscle area of hip, thigh, and lower leg. MRI scans were performed by an experienced radiologist using standard array coils at the parallel part of the abdomen between the L4 and L5 vertebrae in the supine position, the section of the femoral head, the root of thigh, the midpoint of the line between the anterior superior iliac spine and the superior edge of the patella, and the thickest level of the lower leg. Images were segmented and calculated for VFA, ASFA, HSFA, U‐TSFA, M‐TSFA, LSFA, HMA, U‐TMA, M‐TMA, and LMA using SliceOmatic image analysis software (version 4.2; Tomovision Inc., Canada) (shown in Figure 1).

Liver fat was measured by MRI‐PDFF. All scans and measurements were performed by an experienced radiologist. The fat fraction of region of interest in the right anterior, right posterior, and left lobe regions was measured, avoiding major vessels and bile ducts. Then, the average of the three PDFF values was calculated as the liver fat.

### 2.5 Flash glucose monitoring

The FGM system (FreeStyle Libre H, Abbott) was installed in participants to record interstitial glucose levels for 14 consecutive days. The sensor was inserted into the subcutaneous tissue of upper arm. Glucose concentrations were automatically stored every 15 min. According to our pre‐experiment, the best working state of FGM is the middle 10 days, thus sensor data on days 1, 2, 13, and 14 were not included, during which the sensors were installed and the data were inaccurate and incomplete. Key metrics were calculated from the FGM data, including MSG, SDSG, CV, and TIR. SDSG and CV are widely used to quantify glycemic variability.^5^

### 2.6 Three‐dimensional accelerometer test

Three‐dimensional accelerometer tester (wGT3x‐BT, Manufacturing Technology Inc, MTI, Florida, USA) was used to assess the amount of physical activity. Participants were asked to wear accelerometer on the abdomen for 7 days after the baseline assessment, except sleeping, bathing, or other water activities. The amount of physical activity was quantified as minutes and percentages of sitting, low‐, moderate‐ and high‐intensity physical activity using the ActiLife Data Analysis Platform (version 6.13, Manufacturing Technology Inc, Florida, USA).

### 2.7 Statistical analyses

Statistical analyses were performed using SPSS 25.0 software (SPSS, Inc, Chicago, IL) and Prism 7.00 software (GraphPad, San Diego, CA, USA). Kolmogorov–Smirnov test was used to determine the normal distribution of data. Data were expressed as mean ± SD, median (interquartile range), and percentage appropriately. Data with skewed distribution were natural logarithmically transformed (log_e_‐transformed) before analysis. Baseline characteristics were compared between groups by Student's unpaired *t* test for continuous data and chi‐square test for categorical data, respectively. Paired *t* test was used to analyze within‐group changes. A two‐way repeated‐measures analysis of variance was used to examine the changes in blood glucose levels and the interaction effect of time and intervention, with Bonferroni post hoc test adjusting for multiple comparisons. Glycemic variability of participants was assessed by SDSG and CV. Correlations between baseline characteristics and changes in glycemic variability were analyzed using Pearson and partial correlation analysis. Moreover, stepwise multivariable linear regression analysis was conducted to explore the factors that influence on changes in glycemic variability. All statistical tests were two tailed, with a 0.05 significance level.

## 3 RESULTS

### 3.1 Baseline characteristics of study participants

A total of 50 diabetes patients were included and randomized to exercise (*n* = 25) or control (*n* = 25) group. Participants' characteristics are shown in Table 1. The proportion of male participants was 68.0% (*n* = 17) and 52.0% (*n* = 13) for exercise and control group, respectively (*p* = .248). No significant differences in the baseline characteristics, including duration of diabetes, age, body mass index (BMI), waist, blood pressure, body fat, glucose and lipid metabolic indicators, liver and renal function, and MRI‐quantified fat and muscle distribution, were observed between the two groups (Table 1).

**TABLE 1:** Characteristics of study participants between control and exercise group.

Characteristics	Control group (*n* = 25)	Exercise group (*n* = 25)	*p*
Male sex (%)	52.0%	68.0%	.248
Duration of diabetes (month)	61.00 ± 49.06	72.58 ± 54.38	.437
Age (years)	46.56 ± 8.04	48.12 ± 9.04	.522
BMI (kg/m^2^)	27.11 ± 2.81	27.15 ± 3.09	.967
Waist circumference (cm)	92.28 ± 7.21	92.74 ± 6.64	.815
Hip circumference (cm)	98.12 ± 5.27	96.60 ± 5.55	.325
Thigh circumference (cm)	48.62 ± 3.09	47.44 ± 3.92	.243
Systolic blood pressure (mmHg)	122.70 ± 16.87	120.96 ± 13.76	.691
Diastolic blood pressure (mmHg)	77.40 ± 10.29	76.80 ± 9.25	.829
Fat mass (kg)	22.12 ± 5.16	21.55 ± 4.80	.689
Body fat percentage (%)	28.76 ± 5.55	28.20 ± 4.78	.708
HbA1c (%)	7.03 ± 0.45	7.03 ± 0.48	.976
Fasting plasma glucose (mmol/L)	6.77 ± 1.35	6.94 ± 1.61	.692
Fasting insulin (uU/ml)^a^	11.02 (7.38–20.03)	13.18 (9.59–17.02)	.765
HOMA‐IR^§^	3.81 (2.36–5.44)	4.08 (3.35–6.57)	.702
HOMA‐β^§^	70.85 (49.95–130.86)	78.50 (53.37–139.14)	.966
Alanine aminotransferase (U/L)^a^	33.00 (22.00–47.00)	28.50 (21.75–57.25)	.441
Aspartate aminotransferase (U/L)^a^	23.00 (18.00–28.00)	22.00 (18.00–31.75)	.341
γ‐glutamyl transpeptidase (U/L)^a^	30.00 (22.00–52.00)	30.00 (23.50–55.50)	.829
Blood urea nitrogen (mmol/L)^a^	5.40 (4.70–6.35)	5.45 (5.20–6.33)	.964
Serum creatinine (umol/L)^a^	65.90 (54.05–79.85)	72.85 (64.83–78.78)	.219
Serum uric acid (umol/L)	349.79 ± 93.36	366.09 ± 79.52	.524
Total cholesterol (mmol/L)	4.73 ± 1.00	5.14 ± 0.97	.147
Triglycerides (mmol/L)^a^	1.56 (1.18–2.37)	1.71 (1.07–2.39)	.732
HDL‐C (mmol/L)^a^	0.96 (0.80–1.20)	0.96 (0.83–1.10)	.855
LDL‐C (mmol/L)^a^	2.72 (2.34–3.53)	3.15 (2.73–4.04)	.064
SFA (cm^2^)^a^	214.40 (161.05–267.10)	190.70 (163.25–267.15)	.681
VFA (cm^2^)^a^	128.80 (102.90–148.25)	135.40 (115.60–151.35)	.522
HSFA (cm^2^)^a^	205.20 (176.70–248.00)	196.30 (172.15–230.75)	.683
HMA (cm^2^)^a^	273.40 (243.90–296.05)	259.40 (224.00–296.00)	.387
LSFA (cm^2^)^a^	41.17 (38.08–48.05)	39.96 (35.05–43.40)	.260
LMA (cm^2^)^a^	126.90 (114.50–146.85)	119.60 (103.85–152.65)	.644
U‐TSFA (cm^2^)^a^	190.00 (139.35–213.85)	178.40 (143.65–189.15)	.860
U‐TMA (cm^2^)^a^	264.20 (227.00–315.70)	263.60 (210.65–301.00)	.545
M‐TSFA (cm^2^)^a^	95.62 (75.35–125.95)	92.29 (80.34–113.30)	.849
M‐TMA (cm^2^)^a^	238.70 (213.95–280.25)	240.30 (198.50–277.40)	.791
Liver fat (%)^a^	11.48 (6.40–15.57)	9.23 (4.73–14.15)	.495

*Note*: Data are mean ± SD or median (interquartile range).

Abbreviations: ASFA, abdominal subcutaneous fat area; BMI, body mass index; HbA1c, glycated hemoglobin; HDL‐C, high density lipoprotein cholesterol; HOMA, homeostasis model assessment; HMA, hip muscle area; HSFA, hip subcutaneous fat area; LDL‐C, low density lipoprotein cholesterol; LMA, lower leg muscle area; LSFA, lower leg subcutaneous fat area; M‐TMA, mid‐thigh muscle area; M‐TSFA, mid‐thigh subcutaneous fat area; U‐TSFA, upper thigh subcutaneous fat area; U‐TMA, upper thigh muscle area; VFA, visceral fat area.

^a^ Log_e_‐transformed before analysis.

In addition, there was no significant difference in the level of physical activity between the exercise and control groups at baseline (Supplementary Table S1). During the intervention period, the variation in physical activity between the groups was the duration of exercise training, which was specifically provided to the exercise group but not to the control group. Total daily energy intake of the control group was 1697.63 ± 36.43 kcal before the intervention, and it decreased to 1588.97 ± 35.59 kcal during the intervention period. Total daily energy intake of the exercise group was 1677.58 ± 42.07 kcal before the intervention, and it decreased to 1562.72 ± 38.70 kcal during the intervention period. There was no significant difference in energy intakes between the two groups during any study period (Supplementary Tables S1 and S2). Meanwhile, the intakes of carbohydrate, protein, fat, and other nutrients did not differ between the two groups (all *p* > .05).

### 3.2 Exercise improves the blood glucose fluctuation of diabetes patients

**FIGURE 2:**
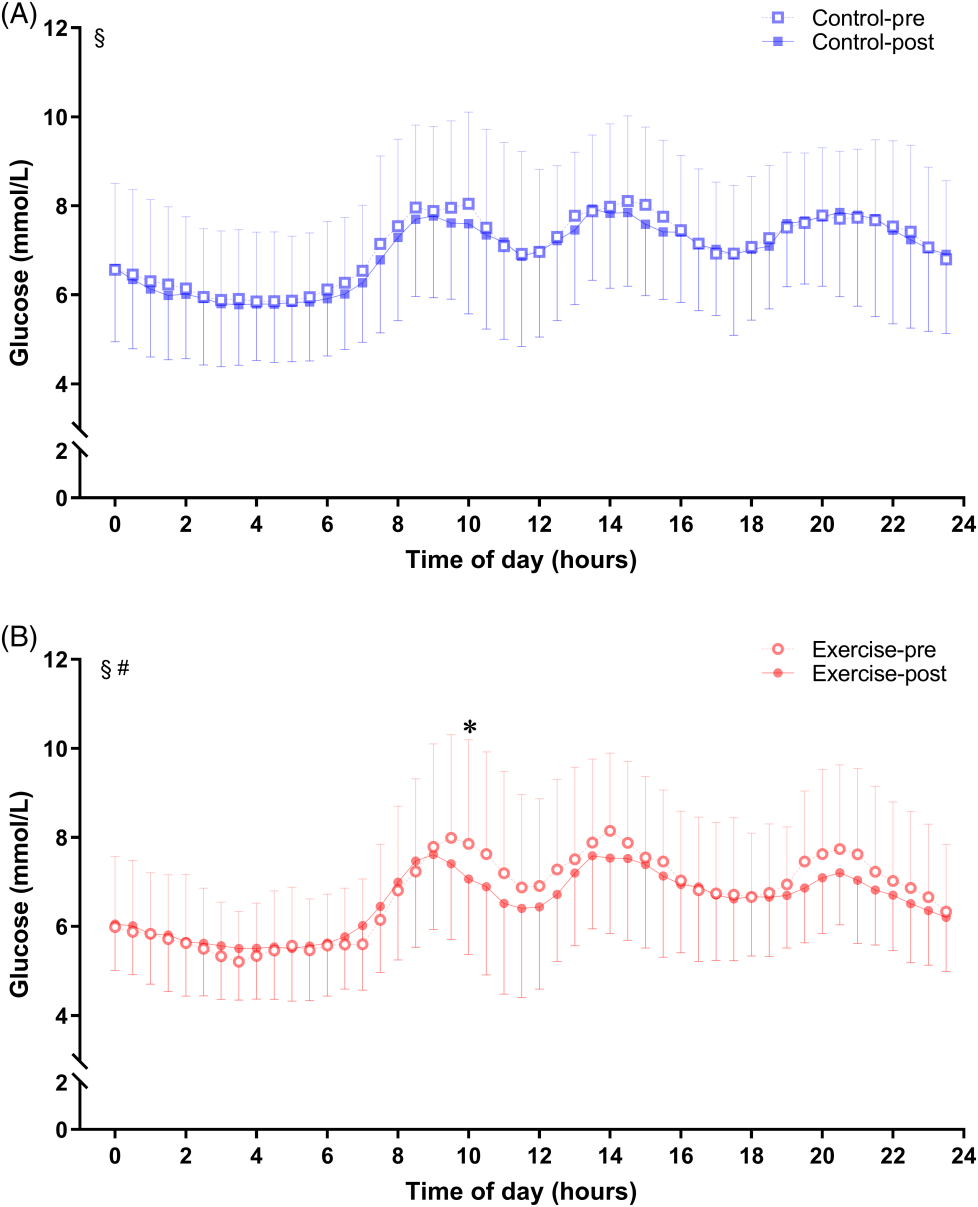
FGM‐based glucose levels in response to study intervention. FGM‐based glucose levels were assessed before and during the intervention. **A,** Blue lines and symbols, control group (*n* = 25); **B,** red lines and symbols, exercise group (*n* = 25). Using two‐way repeated‐measures analysis of variance: ^§^ *p* < .05 for the effect of time; ^#^ *p* < .05 for the interaction between study intervention and time. Using Bonferroni's multiple comparison test: *p < .05 for the difference between pre and post period in each group. Values are mean ± SD. FGM, flash continuous glucose monitor.

The FGM‐based 24 h glucose fluctuations of the two groups before and during the intervention are shown in Figure 2. There were no significant changes in 24 h glucose levels in the control group before and during the intervention. However, a decreasing trend in glucose concentrations was observed in the exercise group. The 24 h glucose curve of the exercise group was more stable and below its baseline curve. Exercise intervention also decreased FGM‐based glucose concentrations compared with the pretraining period at several time points.

**FIGURE 3:**
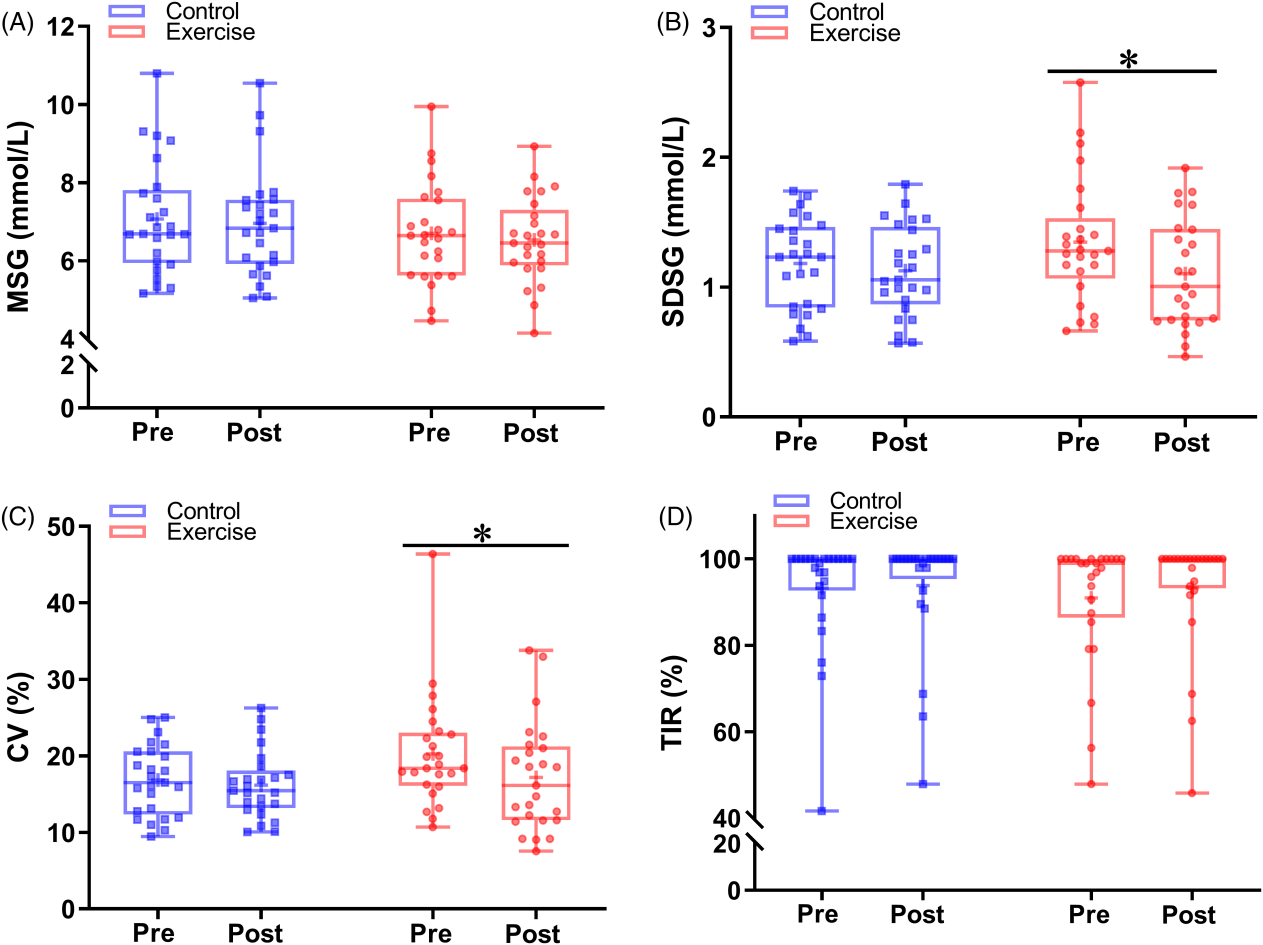
Comparison of glycemic variability indexes between control and exercise group. (A) MSG, (B) SDSG, (C) CV, and (D) TIR levels between pre and post period in the control and exercise groups. Blue lines and symbols, control group (*n* = 25); red lines and symbols, exercise group (*n* = 25). *p < .05 for the difference between pre and post period in each group. CV, coefficient of variation; MSG, mean sensor glucose; SDSG, SD of sensor glucose; TIR, time in range.

Glucose fluctuation was further assessed by calculating MSG, SDSG, CV, and TIR from the FGM data (Figure 3). Baseline MSG (exercise vs control, mean 6.70 vs 7.07 mmol/L, *p* = .343), SDSG (exercise vs control, mean 1.35 vs 1.18 mmol/L, *p* = .172), CV (exercise vs control, mean 20.25 vs 16.84%, *p* = .053), and TIR (exercise vs. control, mean 90.96 vs 93.25%, *p* = .563) did not differ between the two groups. Meanwhile, the levels of these variables were similar in the two groups during the intervention period (all *p* > .05). Significantly, SDSG levels were reduced in exercise group during the training period (exercise‐pre vs exercise‐post, mean 1.35 vs 1.10 mmol/L, *p* = .006), as well as CV (exercise‐pre vs exercise‐post, mean 20.25 vs 17.20%, *p* = .027). No significant changes were observed in the control group with respect to SDSG and CV (all *p* > .05).

### 3.3 Correlations between baseline characteristics and changes in glycemic variability

Glycemic variability of participants was assessed by SDSG and CV. Correlations between baseline characteristics and changes in SDSG and CV (post‐training minus pre‐training, SDSG change and CV change) in the exercise group are shown in Table 2. VFA was significantly correlated with SDSG change (*r* = −0.526, *p* = .007) and CV change (*r* = −0.458, *p* = .021) (Table 2 and Supplementary Figure S1), which remained significant when further adjusted for age and sex (all *p* < .001). However, there was no significant correlation between other baseline variables and changes in SDSG or CV (all *p* > .05). Additionally, the correlations between baseline characteristics and changes in glycemic variability were also analyzed in the control group. As shown in Supplementary Table S3, baseline age was significantly associated with CV change (*r* = −0.463, *p* = .020) but not with SDSG change (*r* = −0.391, *p* = .053) in the control group. Similar to the exercise group, no other baseline parameters were correlated with changes in SDSG and CV in the control group (all *p* > .05).

**TABLE 2:** Associations between baseline characteristics and changes in glycemic variability metrics in exercise group.

Characteristics	SDSG change				CV change			
	Unadjusted		Adjusted for age and sex		Unadjusted		Adjusted for age and sex	
	*r*	*p*	*r*	*p*	*r*	*p*	*r*	*p*
Male sex	−0.048	.821	–	–	0.012	.955	–	–
Age	−0.272	.189	–	–	−0.227	.276	–	–
BMI	−0.190	.364	−0.269	.226	−0.260	.209	−0.371	.090
Fat mass	−0.112	.593	−0.126	.576	−0.215	.302	−0.255	.253
Fat percentage	−0.172	.410	−0.122	.589	−0.289	.161	−0.277	.213
HbA1c	−0.252	.224	−0.215	.336	−0.070	.739	−0.011	.960
FPG	−0.308	.134	−0.299	.177	−0.146	.487	−0.141	.530
HOMA‐IR^a^	−0.331	.106	−0.418	.053	−0.255	.218	−0.404	.062
HOMA‐β^a^	−0.077	.715	−0.137	.543	−0.146	.486	−0.264	.235
Total cholesterol	−0.119	.571	−0.208	.353	0.029	.890	−0.079	.725
Triglycerides^a^	0.143	.505	0.066	.771	0.316	.132	0.284	.200
HDL‐C^a^	−0.238	.252	−0.145	.520	−0.303	.141	−0.254	.253
LDL‐C^a^	−0.093	.659	−0.067	.766	0.014	.949	0.044	.846
ASFA^a^	0.002	.992	0.087	.701	−0.108	.606	−0.049	.828
VFA^a^	−0.526	.007	−0.751	< .001	−0.458	.021	−0.774	< .001
HSFA^a^	0.073	.729	0.105	.641	−0.072	.732	−0.088	.696
HMA^a^	−0.075	.720	−0.330	.133	−0.016	.938	−0.290	.190
LSFA^a^	0.018	.931	−0.041	.855	−0.102	.629	−0.184	.412
LMA^a^	−0.030	.886	−0.231	.300	0.002	.991	−0.239	.283
U‐TSFA^a^	−0.007	.974	0.027	.907	−0.154	.462	−0.127	.572
U‐TMA^a^	−0.009	.966	−0.289	.191	0.034	.871	−0.259	.245
M‐TSFA^a^	0.099	.639	0.097	.668	−0.013	.950	−0.028	.903
M‐TMA^a^	0.053	.802	−0.175	.435	0.118	.574	−0.125	.579
Liver fat^a^	−0.048	.818	−0.110	.626	0.118	.575	0.032	.887

Abbreviations: ASFA, abdominal subcutaneous fat area; BMI, body mass index; CV, coefficient of variation; FPG, fasting plasma glucose; HbA1c, glycated hemoglobin; HDL‐C, high density lipoprotein cholesterol; HOMA, homeostasis model assessment; HMA, hip muscle area; HSFA, hip subcutaneous fat area; LDL‐C, low density lipoprotein cholesterol; LMA, lower leg muscle area; LSFA, lower leg subcutaneous fat area; M‐TMA, mid‐thigh muscle area; M‐TSFA, mid‐thigh subcutaneous fat area; SDSG, SD of sensor glucose; U‐TSFA, upper thigh subcutaneous fat area; U‐TMA, upper thigh muscle area; VFA, visceral fat area.

^a^ Log_e_‐transformed before analysis.

### 3.4 Baseline fat and muscle distribution were determinants of changes in glycemic variability

We further investigated the factors that influence on changes in glycemic variability in the exercise group, using stepwise multiple linear regression. Baseline age, BMI, fat mass, fat percentage, HbA1c, FPG, HOMA‐IR, HOMA‐β, ASFA, VFA, HSFA, HMA, U‐TSFA, U‐TMA, M‐TSFA, M‐TMA, LSFA, and LMA were input to the model. The contributions of all significant factors in the final model are shown in Table 3. Baseline VFA (β = −0.761, *p* = .001) and M‐TMA (β = 0.450, *p* = .027) were significantly independent predictors of SDSG change, and explained 37.2% of the variance. Meanwhile, baseline VFA (β = −0.637, *p* < .001) and BMI (β = −1.096, *p* < .001) were negatively associated with CV change, and M‐TMA (β = 1.274, *p* < .001) and HSFA (β = 0.594, *p* = .004) were positively associated with CV change, accounting for 62.2% of the variance. Accordingly, baseline MRI‐quantified fat and muscle distribution, including VFA and M‐TMA, were independent determinants of changes in glycemic variability (all *p* < .05).

**TABLE 3:** Stepwise multivariable linear regression for changes in glycemic variability metrics in exercise group.

Glycemic variability	Variable	B	SEr	β	*p*	F	*p*	Adjusted *R* ^2^
SDSG change	VFA^§^	−0.902	0.225	−0.761	.001	8.096	.002	0.372
	M‐TMA^§^	0.797	0.336	0.450	.027			
CV change	VFA^§^	−11.988	2.809	−0.637	< .001	10.856	< .001	0.622
	M‐TMA^§^	35.854	6.562	1.274	< .001			
	BMI	−2.288	0.524	−1.096	< .001			
	HSFA^§^	18.123	5.627	0.594	.004			

*Note*: Multivariable linear regression model includes age, BMI, fat mass, fat percentage, HbA1c, FPG, HOMA‐IR, HOMA‐β, ASFA, VFA, HSFA, HMA, U‐TSFA, U‐TMA, M‐TSFA, M‐TMA, LSFA, and LMA.

Abbreviations: ASFA, abdominal subcutaneous fat area; BMI, body mass index; CV, coefficient of variation; FPG, fasting plasma glucose; HbA1c, glycated hemoglobin; HOMA, homeostasis model assessment; HMA, hip muscle area; HSFA, hip subcutaneous fat area; LMA, lower leg muscle area; LSFA, lower leg subcutaneous fat area; M‐TMA, mid‐thigh muscle area; M‐TSFA, mid‐thigh subcutaneous fat area; SDSG, SD of sensor glucose; U‐TSFA, upper thigh subcutaneous fat area; U‐TMA, upper thigh muscle area; VFA, visceral fat area.

## 4 DISCUSSION

In this randomized controlled trial, combined exercise training improved blood glucose fluctuation, with reduction in SDSG and CV levels. Notably, the body compositions of participants were accurately quantified using MRI and MRI‐PDFF, and baseline fat and muscle distribution were independent and significant factors of changes in glycemic variability metrics. Patients with greater abdominal obesity and lower thigh circumference had better improvements in glycemic variability after the 1‐week combined training. Our results showed that baseline phenotypes affected the glycemic response to exercise in type 2 diabetes patients.

In the present study, no significant changes in mean glucose levels and TIR were observed after the combined aerobic and resistance exercise training. Winding et al^30^ reported that 11 weeks of endurance training induced a significant decrease in mean CGM glucose levels and TIR. Therefore, the relatively short duration of exercise training may partially account for it in our study. Nevertheless, our results showed that glycemic variability was significantly improved during the exercise training period. Minnock et al conducted a randomized controlled clinical study in 12 patients with type 1 diabetes to compare the effect of acute aerobic, resistance, and combined exercise training (40 min each) on interstitial glucose variability, which was assessed using mean amplitude of glycemic excursions, SD, and CV. The results showed that combined exercise was more effective to improve glucose fluctuations,^16^ which is consistent with our findings. Cai et al^31^ also reported that 3 weeks of moderate‐to‐vigorous exercise (≥30 min per day) decreased CV in people with obesity. Dawn phenomenon refers to a spontaneous hyperglycemia or an increased demand for insulin to maintain normoglycemia in the early morning, which affects nearly half of the diabetes patient population.^32^, ^33^ Zheng et al found that 3 consecutive days of acute moderate‐intensity aerobic exercise before breakfast could decrease the morning blood glucose levels, reduce blood glucose fluctuations, and improve glycemic control throughout the day in type 2 diabetes patients with dawn phenomenon.^34^ Exercise‐stimulated glucose uptake is crucial for the regulation of glycemic control.^23^ Additionally, exercise has been shown to reduce inflammation and protect tissues from oxidative stress, both of which play a role in the pathogenesis of diabetes.^35^ Taken together, even acute or short‐term exercise is sufficient to improve blood glucose fluctuation, whereas the improvement in mean glucose levels needs greater volumes and longer duration of exercise training.

The number of studies dealing with regular physical activity and exercise training and their effects on metabolic indicators and cardiorespiratory fitness is growing. Notably, most of these studies reported not only metabolic improvements in response to exercise but also considerable individual differences.^36^, ^37^, ^38^, ^39^ In our study, the overall glycemic variability metrics levels were decreased after exercise training. However, not all participants benefit from exercise training, a greater range and even increases in SDSG and CV levels were also observed, which suggested the heterogeneity in glycemic response. The exact mechanisms or determinants responsible for the heterogeneity in response to exercise are unclear. Some studies have reported that the baseline characteristics of a sedentary state had a considerable impact on the responsiveness.^40^ We investigated the association between baseline parameters and changes in SDSG and CV in the current study and found that baseline visceral fat area, midthigh muscle area, and BMI significantly influenced the glycemic response to exercise, with better improvements of glycemic variability observed in the patients with more visceral fat and less thigh muscle mass. These results were consistent with previous studies reporting that type 2 diabetes patients with poorer baseline body composition and higher baseline blood glucose and HbA1c were more likely to achieve an improvement in glycemic control after exercise intervention.^36^, ^39^, ^41^ Higher BMI, body fat, and insulin resistance were associated with higher MSG and CV levels.^42^ Thus, the changes in CV and SDSG were more likely to be greater in patients with abdominal obesity after exercise training. Moreover, the type and amount of food consumed are also important factors that influence on blood glucose fluctuation.^6^ Low‐carbohydrate diets can improve glucose homeostasis in people with obesity, with decline in serum glycated albumin, MSG, and CV.^31^ During the study period, we required all participants to prepare and consume their meals according to the Dietary Guidelines for Chinese Residents. Patients with abdominal obesity had an eating behavior of consuming more high‐fat and high‐carbohydrate products at baseline, so their dietary intakes may change more after the study intervention. Thus, the impact of dietary intervention on blood glucose excursions may partially account for better glycemic response to exercise in patients with greater abdominal obesity.

To our knowledge, this is the first clinical trial to investigate the role of MRI‐quantified body composition in exercise response reflected by glucose fluctuation. We reported that type 2 diabetes patients with greater abdominal obesity and lower thigh circumference could benefit from exercise training, with better improvement of glycemic variability. Meanwhile, this study found that even lower intensity of exercise regimes was sufficient to improve glycemic variability. Some limitations should also be considered in this study. First, this trial demonstrated the effects of very short‐term exercise training on glucose fluctuation. Studies of longer training duration are needed to investigate the persistence of blood glucose response. Second, considering the relatively small sample size of this trial, the findings should be interpreted with caution. Our encouraging findings provide new insights into individualized exercise intervention for diabetes patients. Further prospective clinical trials are warranted to validate the correlation between body composition and glycemic response to exercise in type 2 diabetes.

In conclusion, this study reported that acute combined aerobic and resistance exercise training could improve glycemic fluctuation in type 2 diabetes patients. Baseline MRI‐quantified fat and muscle distribution were significant determinants of changes in glycemic variability. Findings from our study revealed the role of body composition in glycemic response to exercise, providing new insights into personalized exercise intervention for diabetes patients.

## AUTHOR CONTRIBUTIONS

Dan Liu performed statistical analyses and wrote the manuscript. Ying Zhang researched the data and wrote the manuscript. Qian Wu, Rui Han, Di Cheng, and Liang Wu critically reviewed and edited the manuscript. Jingyi Guo, Xiangtian Yu, Wenli Ge, Jiacheng Ni, Yaohui Li, Tianshu Ma, Qichen Fang, Yufei Wang, Yan Zhao, Yanan Zhao, and Biao Sun helped in statistical analysis and data collection and critically reviewed the manuscript. Huating Li and Weiping Jia conceptualized the study concept and design and edited the manuscript. All authors have read and approved the final manuscript.

## FUNDING INFORMATION

This work was supported by the National Key Research and Development Program of China (2022YFA1004804) to Weiping Jia and Huating Li; the Excellent Young Scholars of NSFC (82022012), General Program of NSFC (81870598), Two Hundred Program from Shanghai Jiao Tong University School of Medicine (20191830), and Innovative research team of high‐level local universities in Shanghai (SHSMU‐ZDCX20212700) to Huating Li; the Shanghai Municipal Key Clinical Specialty (2017ZZ01013), Shanghai Key Clinical Specialty Construction Project (LY01.05.02), the National Key Research and Development Program of China (2018YFA0800402), and Shanghai Research Center for Endocrine and Metabolic Diseases (2022ZZ01002) to Weiping Jia; the National Natural Science Foundation of China (82100879), Shanghai Pujiang Program (2020PJD044), and Exploration Fund Grant of Shanghai Sixth People's Hospital (ynts202003) to Liang Wu.

## CONFLICT OF INTEREST STATEMENT

All the authors have no conflicts of interest to declare.

## Supporting information


**Table S1.** Physical activity levels and energy intake between control and exercise group at baseline.
**Table S2.** Energy intake between exercise and control group during study intervention.
**Table S3.** Associations between baseline characteristics and changes in glycemic variability metrics in control group.
**Figure S1.** Correlations of (A) SDSG change, (B) CV change with baseline VFA in exercise group. Abbreviations: CV, coefficient of variation; SDSG, SD of sensor glucose; VFA, visceral fat area.

## Data Availability

Restrictions apply to the availability of data generated or analyzed during this study to preserve patient confidentiality or because they were used under license. Data are, however, available from the authors upon reasonable request.
