# Supplementary material for: Exercise‐induced improvement of glycemic fluctuation and its relationship with fat and muscle distribution in type 2 diabetes
Source: J Diabetes. 2024 Apr 7;16(4):e13549. doi: 10.1111/1753-0407.13549 (PMC10999499; doi:10.1111/1753-0407.13549)
Supplement: Supplementary file 1 — Table S1. Physical activity levels and energy intake between control and exercise group at baseline. Table S2. Energy intake between exercise and control group during study intervention. Table S3. Associations between baseline characteristics and changes in glycemic variability metrics in control group. Figure S1. Correlations of (A) SDSG change, (B) CV change with baseline VFA in exercise group. Abbreviations: CV, coefficient of variation; SDSG, SD of sensor glucose; VFA, visceral fat area. [file JDB-16-e13549-s001.docx]

**Supplementary Material**

**Supplementary Table S1** Physical activity levels and energy intake between control and exercise group at baseline

| **Characteristics** | **Control group (n=25)** | **Exercise group (n=25)** | ***p*** |
| --- | --- | --- | --- |
| **Physical activity** |  |  |  |
| Sitting (min/day) | 661.76 ± 56.43 | 626.76 ± 42.54 | .623 |
| Mild physical activity (min/day) | 108.13 ± 12.08 | 179.74 ± 47.30 | .149 |
| Moderate-to-vigorous physical activity (min/day) | 26.04 ± 4.32 | 33.33 ± 3.81 | .211 |
| **Dietary intake** |  |  |  |
| Energy intake (kcal/day) | 1697.63 ± 36.43 | 1677.58 ± 42.07 | .720 |
| Carbohydrate (g/day) | 209.56 ± 5.81 | 204.35 ± 7.20 | .576 |
| Protein (g/day) | 75.17 ± 2.99 | 80.35 ± 4.52 | .345 |
| Fat (g/day) | 64.80 ± 3.26 | 62.74 ± 3.20 | .654 |
| Cholesterol (mg/day) | 851.57 ± 96.94 | 888.72 ± 118.24 | .809 |
| Fiber (g/day) | 20.88 ± 2.41 | 21.93 ± 2.54 | .767 |
| Vitamin A (µg/day) | 800.54 ± 105.40 | 1288.67 ± 488.68 | .334 |
| Vitamin B1 (mg/day) | 1.78 ± 0.23 | 1.67 ± 0.23 | .738 |
| Vitamin B2 (mg/day) | 0.75 ± 0.05 | 0.95 ± 0.09 | .060 |
| Vitamin B3 (mg/day) | 31.78 ± 3.04 | 30.20 ± 3.57 | .738 |
| Vitamin C (mg/day) | 122.63 ± 27.38 | 146.66 ± 24.75 | .518 |
| Vitamin E (mg/day) | 18.71 ± 1.36 | 24.91 ± 3.23 | .083 |
| Na (mg/day) | 3162.04 ± 297.06 | 3851.63 ± 303.09 | .111 |
| Ca (mg/day) | 729.52 ± 77.49 | 930.10 ± 90.90 | .100 |
| Fe (mg/day) | 29.29 ± 2.46 | 28.31 ± 2.28 | .771 |

*Note*: Data are mean ± SEM.

**Supplementary Table S2** Energy intake between exercise and control group during study intervention

| **Characteristics** | **Control group (n=25)** | **Exercise group (n=25)** | ***p*** |
| --- | --- | --- | --- |
| Energy intake (kcal/day) | 1588.97 ± 35.59 | 1562.72 ± 38.70 | .620 |
| Carbohydrate (g/day) | 200.71 ± 3.78 | 193.81 ± 5.62 | .314 |
| Protein (g/day) | 76.15 ± 3.40 | 78.84 ± 2.75 | .540 |
| Fat (g/day) | 55.47 ± 2.18 | 53.72 ± 1.95 | .553 |
| Cholesterol (mg/day) | 377.97 ± 51.41 | 535.01 ± 93.78 | .149 |
| Fiber (g/day) | 11.43 ± 1.11 | 11.60 ± 0.95 | .905 |
| Vitamin A (µg/day) | 461.97 ± 49.26 | 621.07 ± 204.05 | .452 |
| Vitamin B1 (mg/day) | 0.95 ± 0.13 | 0.87 ± 0.08 | .609 |
| Vitamin B2 (mg/day) | 1.00 ± 0.10 | 1.02 ± 0.08 | .867 |
| Vitamin B3 (mg/day) | 4.81 ± 1.46 | 6.56 ± 2.32 | .525 |
| Vitamin C (mg/day) | 93.97 ± 12.55 | 94.13 ± 12.83 | .993 |
| Vitamin E (mg/day) | 18.65 ± 2.65 | 16.06 ± 1.72 | .415 |
| Na (mg/day) | 2401.53 ± 209.49 | 2530.66 ± 184.18 | .645 |
| Ca (mg/day) | 605.24 ± 72.76 | 602.52 ± 56.76 | .977 |
| Fe (mg/day) | 17.45 ± 1.26 | 17.50 ± 2.18 | .985 |

*Note*: Data are mean ± SEM.

**Supplementary Table S3** Associations between baseline characteristics and changes in glycemic variability metrics in control group

| **Characteristics** | **SDSG change** | | | |  | **CV change** | | | |
| --- | --- | --- | --- | --- | --- | --- | --- | --- | --- |
|  | **Unadjusted** | | **Adjusted for age and sex** | |  | **Unadjusted** | | **Adjusted for age and sex** | |
|  | ***r*** | ***p*** | ***r*** | ***p*** |  | ***r*** | ***p*** | ***r*** | ***p*** |
| Male | -0.067 | .752 | — | — |  | -0.100 | .635 | — | — |
| Age | -0.391 | .053 | — | — |  | -0.463 | .020 | — | — |
| BMI | 0.047 | .823 | -0.099 | .652 |  | 0.364 | .074 | 0.251 | .247 |
| Fat mass | 0.001 | .996 | -0.120 | .584 |  | 0.213 | .306 | 0.124 | .574 |
| Fat percentage | -0.057 | .787 | -0.113 | .608 |  | 0.006 | .978 | 0.044 | .844 |
| HbA1c | -0.175 | .402 | -0.344 | .108 |  | -0.157 | .452 | -0.327 | .127 |
| FPG | 0.028 | .894 | 0.025 | .911 |  | 0.075 | .720 | 0.081 | .713 |
| HOMA-IR ^a^ | 0.140 | .505 | 0.032 | .885 |  | 0.256 | .217 | 0.150 | .495 |
| HOMA-β ^a^ | 0.132 | .529 | -0.005 | .983 |  | 0.257 | .216 | 0.112 | .610 |
| Total cholesterol | 0.071 | .736 | 0.044 | .843 |  | 0.042 | .841 | 0.029 | .895 |
| Triglycerides ^a^ | 0.136 | .518 | 0.113 | .608 |  | 0.106 | .613 | 0.072 | .743 |
| HDL-C ^a^ | -0.155 | .460 | -0.050 | .819 |  | -0.215 | .303 | -0.079 | .720 |
| LDL-C ^a^ | 0.128 | .541 | 0.047 | .831 |  | 0.181 | .388 | 0.126 | .567 |
| ASFA ^a^ | 0.093 | .657 | -0.034 | .876 |  | 0.354 | .083 | 0.251 | .247 |
| VFA ^a^ | 0.029 | .892 | 0.021 | .926 |  | 0.116 | .581 | 0.107 | .627 |
| HSFA ^a^ | 0.133 | .525 | -0.067 | .761 |  | 0.232 | .265 | 0.074 | .738 |
| HMA ^a^ | -0.004 | .985 | -0.013 | .952 |  | 0.068 | .746 | 0.039 | .859 |
| LSFA ^a^ | 0.184 | .380 | 0.063 | .774 |  | 0.255 | .219 | 0.173 | .429 |
| LMA ^a^ | 0.127 | .544 | -0.057 | .796 |  | 0.288 | .163 | 0.104 | .636 |
| U-TSFA ^a^ | 0.085 | .686 | -0.098 | .657 |  | 0.153 | .467 | 0.030 | .893 |
| U-TMA ^a^ | 0.121 | .566 | 0.069 | .753 |  | 0.261 | .207 | 0.262 | .228 |
| M-TSFA ^a^ | 0.273 | .187 | 0.155 | .479 |  | 0.244 | .239 | 0.120 | .584 |
| M-TMA ^a^ | 0.170 | .417 | 0.004 | .985 |  | 0.253 | .223 | 0.048 | .829 |
| Liver fat ^a^ | 0.097 | .645 | 0.034 | .878 |  | 0.298 | .147 | 0.272 | .210 |

Abbreviations: ASFA, abdominal subcutaneous fat area; BMI, body mass index; CV, coefficient of variation; FPG, fasting plasma glucose; HDL-C, high density lipoprotein cholesterol; HOMA, homeostasis model assessment; HMA, hip muscle area; HSFA, hip subcutaneous fat area; LDL-C, low density lipoprotein cholesterol; LMA, lower leg muscle area; LSFA, lower leg subcutaneous fat area; M-TMA, mid-thigh muscle area; M-TSFA, mid-thigh subcutaneous fat area; SDSG, SD of sensor glucose; U-TSFA, upper thigh subcutaneous fat area; U-TMA, upper thigh muscle area; VFA, visceral fat area.

^a^ Log_e_-transformed before analysis.


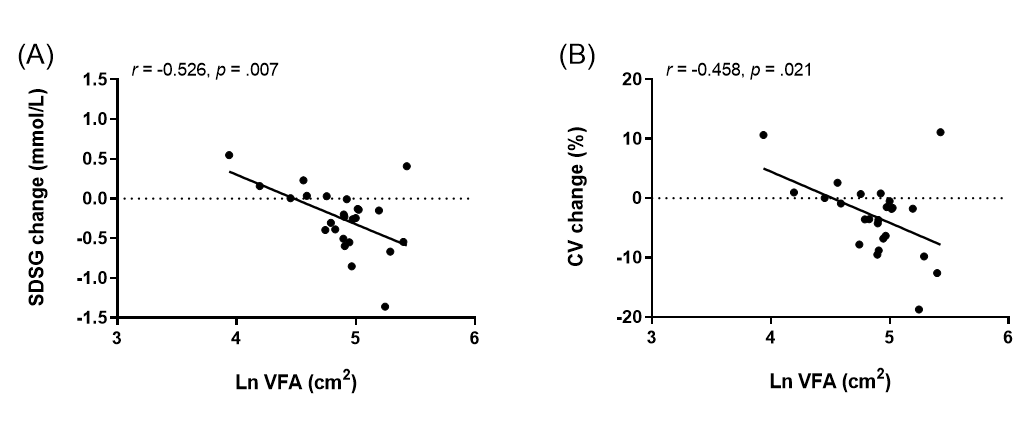


**Supplementary Figure S1** Correlations of (A) SDSG change, (B) CV change with baseline VFA in exercise group. Abbreviations: CV, coefficient of variation; SDSG, SD of sensor glucose; VFA, visceral fat area.
